# Supplementary material for: Host GPCR-cAMP signaling balances Gαs and Gαi activity to control intracellular Brucella infection
Source: bioRxiv. 2026 Jan 6:2026.01.06.697936. Preprint. [Version 1] doi: 10.64898/2026.01.06.697936 (PMC12803190; doi:10.64898/2026.01.06.697936)
Supplement: Supplement 1 [file media-1.pdf]

Supplementary Data

Host GPCR-cAMP signaling balances Gαs and Gαi activity to control intracellular *Brucella* infection

Yoon-Suk Kang[1] & James E. Kirby[1, *]

[1]Department of Pathology, Beth Israel Deaconess Medical Center & Harvard Medical School, Boston, MA, 02215, United States of America

* Corresponding author: James E Kirby

Email: jekirby@bidmc.harvard.edu

Supplementary Tables

Table S1. The effects of Adenosine or dopamine receptor agonists on Bn intracellular growth in THP-1 macrophages.[a]

Target & Functions	Compounds	Z-score
Adenosine A1 receptor agonist	N6-Cyclopentyladenosine; CPA	-7.0 (Strong)
	2-Chloro-N6-cyclopentyladenosine	-4.3 (Weak)
	N6-Cyclohexyladenosine; CHA	-7.1 (Strong)
	SDZ WAG 994	-7.0 (Strong)
	(±)-5'-Chloro-5'-deoxy-ENBA	-7.2 (Strong)
	2'-MeCCPA	-1.2
Adenosine A3 receptor agonist	2-Cl-IB-MECA	-3.1 (Weak)
	IB-MECA	-2.5
	Inosine	-1.9
Adenosine A2A receptor agonist	CGS-21680	-0.3
	Limonene	-2.0
Adenosine A2B receptor agonist	NECA	1.1
Dopamine D1 & D5 receptor agonist	Dihydrexidine	-3.0
	Dopamine	1.9
	Fenoldopam	-0.89
Dopamine D1 receptor agonist	SKF-82958	2.7
	SKF-38393	6.5
	6-Br-APB	-0.17

	A-68930	5.6
	Cabergolin	1.9
	Pergolide	2.0
Dopamine D4 receptor agonist	WAY-100635	-6.3 (Medium)
	ABT 724 trihydrochloride	-7.3(Strong)
	PD 168077 maleate	-6.9 (Medium)
	CP-226269	-1.1
Dopamine D4 receptor antagonist	Clozapine[b]	10.0
Serotonin 5-HT1A receptor agonist	MDL 73005EF hydrochloride	-9.5 (Strong)

[a]Data from Kang & Kirby (1). Z-scores were calculated across screening plates from the primary THP-1 macrophage screen to identify statistically significant inhibitors of intracellular *B. neotomae* growth.

[b]Mixed agonist/antagonist. Strongest effects are as D4 antagonist (2).

Table S2. Phosphodiesterase (PDE) inhibitors affecting Bn intracellular growth in THP-1 macrophages.[a]

Compounds	Primary PDE Inhibitory Activity (reported)	Bn inhibition (% of control)
IBMX	nonselective	-4.8%
Pentoxifyline	nonselective (weak)	-9.8%
Papaverine	nonselective	-27%
Ibudilast	nonselective	-21%
Vinpocetine	PDE1 (additional targets described)	28%
Cilostazol	PDE3	90%
Cilostamide	PDE3	89%
Enoximone	PDE3	83%
Imazodan	PDE3	40%
Milrinone	PDE3	2.4%
Trequinsin	PDE3	45%
Olprinone	PDE3	33%
Quazinone	PDE3	12%
ICI-63197	PDE3/4	20%
YM 976	PDE4	24%
Rolipram	PDE4	-0.7%
Quercetin	PDE4 (pleiotropic flavonoid)	-64%
Sildenafil	PDE5	-3.5%
Zaprinast	PDE5	-0.5%
BRL 50481	PDE7	53%

[a]Data from Kang & Kirby (1). Percent inhibition values reflect normalized intracellular growth relative to vehicle-treated controls and are provided to convey biological effect size.

Table S3. Axenic growth IC_{50} values, host cell cytotoxicity, and maximal rescue of ENBA-mediated inhibition of intracellular *Brucella neotomae* growth by cyclic nucleotide analogs.

	8-Br-cAMP	8-CPT-cAMP	8-Br-cGMP	Dibutyryl-cGMP	cGMP	8-CPT-cGMP
Bn axenic growth IC_{50} (μg/mL)	>50	>50	>50	>50	>50	>50
Host cell CC_{50} (J774A.1, μg/mL)	25	32	>50	16	>50	>50
Maximal fold rescue of intracellular growth	15	10	No rescue	2.3	2.0	No rescue
Concentration alleviating ENBA-mediated inhibition (μg/mL)	21	21	No rescue	9.1	50	No rescue

Data shown correspond to rescue experiments presented in Fig. 3C. Cyclic nucleotide analogs were tested in J774A.1 macrophages infected with *B. neotomae* in the presence of ENBA (2 μg/mL). Maximal fold rescue reflects the increase in intracellular luminescence relative to ENBA-treated controls. Concentrations listed were below axenic growth IC_{50} values and host cell cytotoxicity thresholds.

Table S4. Bacterial strains, cell lines, and primers used in this study.

Bacterial strains		
Strain	*Relevant characteristics*	*Source or Reference*
B. neotomae 5K33	Parent biosafety level 2 rodent pathogen	BEI Resources
B. neotomae-Lux	Transposon mutant of 5K33 expressing Lux operon	(3)
B. neotomae-tdTomato	Transposon mutant of 5K33 having proD/tdtomato-nat genes	
B. neotomae ΔvirB4-Lux	Transposon mutant of virB4 in-frame deletion mutant expression Lux-operon	
B. neotomae ΔvirB4-tdTomato	Transposon mutant of Bn ΔvirB4 having proD/tdtomato-nat genes	
L. pneumophila 02fla-Lux	*L. pneumophila* 02fla having Lux operon	(4)
NEB-5α	*fhuA2 Δ(argF-lacZ)U169 phoA glnV44 Φ80 Δ(lacZ)M15 gyrA96 recA1 relA1 endA1 thi-1 hsdR17*	NEB
Eukaryotes		
Cell line		*Source or Reference*
J774A.1		ATCC TIB-67
THP-1		ATCC TIB-202
Oligonucleotides		
Name	*Sequences*	*Characteristics*
IRES-F	CGC*GGATCC*CCCCTCTCCCTCCCCC	IRES amplification & IRES-eYFP fusion
IRES_eYFP-R	CATCATGGTGGCTTATCATCGTGTTTTCA	
eYFP-F	ATAAGCCACCATGATGGTGAGCAAGGGCGA	eYFP amplification & IRES-eYFP::Gγ fusion
eYFP::Gγ-R	CGC*GAATTC*CTAGAGAATTATGCAAGGCTT	
Gαs-F	CGC*GCGGCCGC*GCCACCATGGGCTGCCTCGG	Gαs subunit amplification & Gαs::NLuc fusion
Gαs::NLuc-R	GGAGCCGCCACCACCGAGCAGCTCGTATTG	
NLuc-F	CTCGGTGGTGGCGGCTCCGTCTTCACACTCGAA	NLuc amplification & Gαs::NLuc fusion
NLuc-R	CGC*GGATCC*TTACGCCAGAATGCGTTCGCA	

Table S5. siRNA used in this study.

Target	Sequences	Use
Adenosine A1 receptor	Sense 5'-AGCAUGGAGUACAUGGUCUACUUCA-3'	Adenosine A1 knockout
	Antisense	

	5’-UGAAGUAGACCAUGUACUCCAUGCUGA-3’	
Dopamine D4 receptor	Sense<br>5’-GCAGACACCCACCAACUACUUCATC-3’	Dopamine D4 knockout
	Antisense<br>5’-GAUGAAGUAGUUGGUGGGUGUCUGCAG-3’	
Non-target siRNA	Sense<br>5′-UUCUCCGAACGUGUCACGU-3′	
	Antisense<br>5′-ACGUGACACGUUCGGAGAA-3′	

Supplementary Figures

A

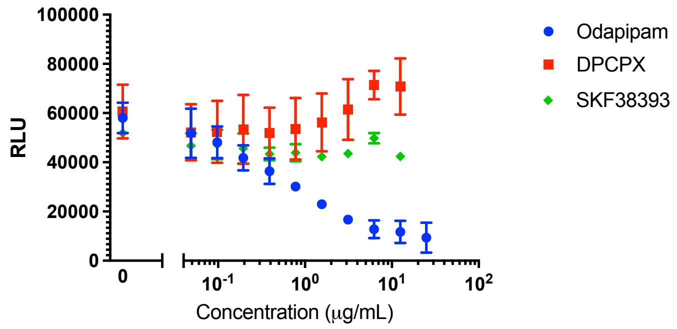

B

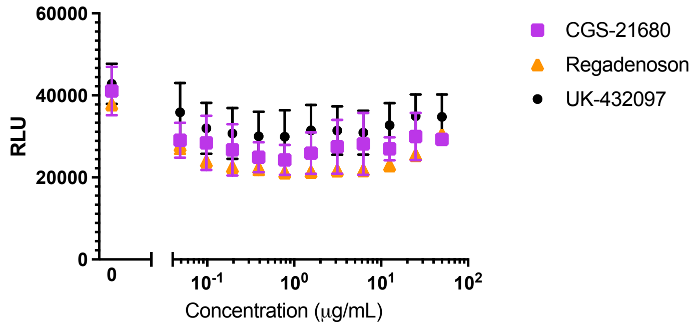

Figure S1. GPCR pathway specificity of host-mediated modulation of *B. neotomae* intracellular growth. (A) Intracellular growth of luminescent *B. neotomae* (Bn) in J774A.1 macrophages measured 48 h post infection following treatment with ligands targeting dopamine and adenosine receptors with distinct G-protein coupling profiles. Odapipam (dopamine D1/D5 receptor antagonist; Gαs-associated), SKF38393 (dopamine D1/D5 receptor agonist; Gαs-associated), and DPCPX (adenosine A1 receptor antagonist; Gαi-associated) were tested over a range of concentrations. **(B)** Intracellular Bn growth measured 48 h post infection following treatment with selective adenosine A2A receptor agonists CGS-21680, regadenoson, and UK-432097, all of which signal predominantly through Gαs. Across the concentrations tested, including the highest concentrations, activation of Gαs-coupled adenosine A2A receptors or dopamine D1/D5 receptors did not significantly alter intracellular Bn growth, whereas antagonism of the Gαs-coupled dopamine D1/D5 receptor with odapipam resulted in dose-dependent inhibition. Data points represent the mean ± standard deviation (SD) calculated from single measurements obtained in two independent experiments. Intracellular bacterial burden is reported as relative luminescence units (RLU).

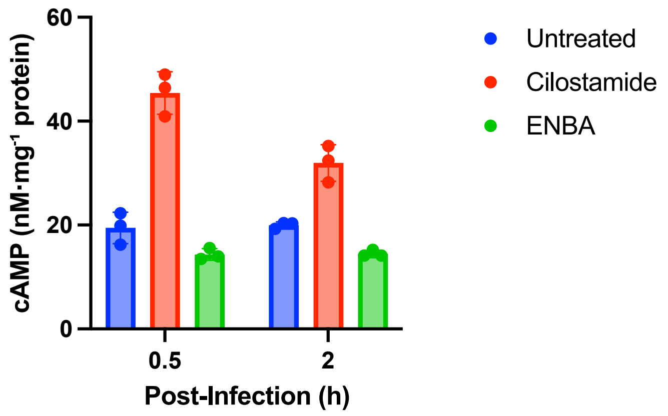

Figure S2. Effects of cilostamide and ENBA on intracellular cAMP levels in uninfected J774A.1 macrophages. Uninfected J774A.1 macrophages were treated with the PDE3 inhibitor cilostamide or the adenosine A_1 receptor agonist ENBA (each at 2 µg/mL), and intracellular cAMP levels were measured after 30 min and 2 h. cAMP concentrations were normalized to total cellular protein. Data represent the mean ± SEM from three independent experiments.

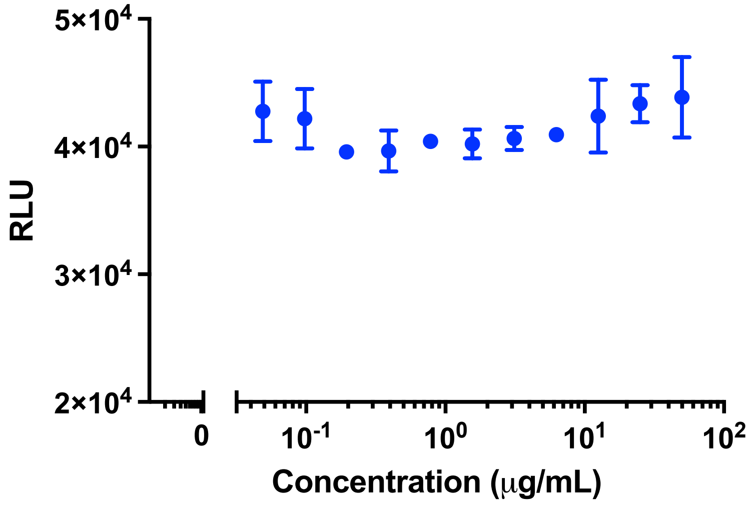

Figure S3. The specific PKA inhibitor PKI 14-22 amide does not affect intracellular *Brucella neotomae* growth. J774A.1 macrophages were infected with luminescent *B. neotomae* and treated with increasing concentrations of the myristoylated PKA inhibitor PKI 14-22 amide. Intracellular bacterial growth was quantified by luminescence 48 h post-infection. No inhibition of intracellular growth was observed, including at the highest concentration tested (50 μg/mL). Data represent the mean ± standard deviation of two independent measurements.

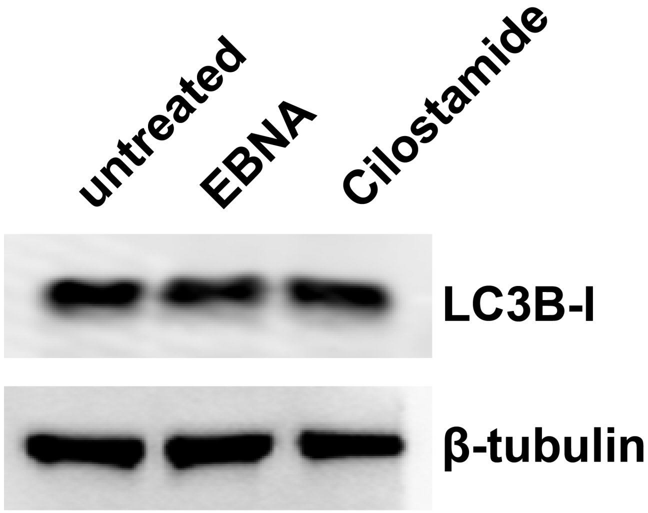

Figure S4. Effects of cilostamide and ENBA on LC3B levels during ΔvirB4 infection of J774A.1 macrophages. J774A.1 macrophages were infected with the type IV secretion system-deficient ΔvirB4 mutant of *Brucella neotomae* and treated with cilostamide or ENBA (2 µg/mL). After 48 h, LC3B levels were assessed by immunoblotting. Only the non-lipidated form, LC3B-I, was detected under these conditions; the lipidated form LC3B-II was not observed. β-tubulin served as a loading control. These findings indicate that modulation of intracellular replication by ENBA or cilostamide is not associated with detectable LC3B lipidation under ΔvirB4 infection conditions.

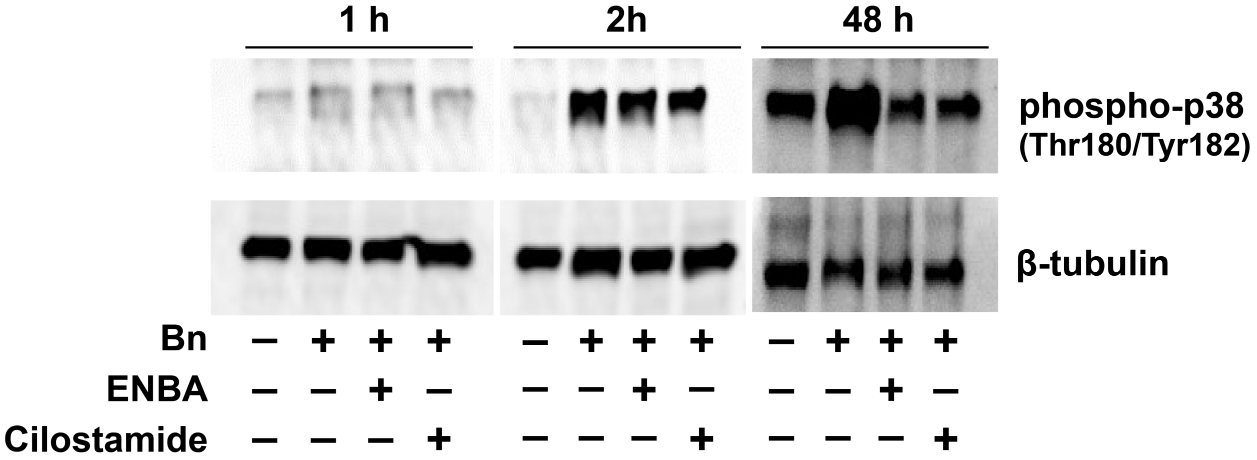

Figure S5. Effects of ENBA and cilostamide on p38 MAP kinase phosphorylation during *Brucella neotomae* infection. J774A.1 macrophages were infected with wild-type *B. neotomae* and treated with ENBA or cilostamide (2 µg/mL), as indicated. Cell lysates were collected at early (1 h and 2 h) and late (48 h) time points post infection, and phosphorylation of p38 MAP kinase was assessed by immunoblotting. β-tubulin served as a loading control. During early infection, p38 phosphorylation was induced by *B. neotomae* infection and was not altered by ENBA or cilostamide treatment. In contrast, at 48 h post infection, p38 phosphorylation was reduced by both ENBA and cilostamide.

Supplementary References

1. Kang YS, Kirby JE. 2019. A Chemical Genetics Screen Reveals Influence of p38 Mitogen-Activated Protein Kinase and Autophagy on Phagosome Development and Intracellular Replication of *Brucella neotomae* in Macrophages. Infect Immun 87:e00044-19.
2. Van Tol HH, Bunzow JR, Guan HC, Sunahara RK, Seeman P, Niznik HB, Civelli O. 1991. Cloning of the gene for a human dopamine D4 receptor with high affinity for the antipsychotic clozapine. Nature 350:610-4.
3. Kang YS, Kirby JE. 2017. Promotion and Rescue of Intracellular *Brucella neotomae* Replication during Coinfection with *Legionella pneumophila*. Infect Immun 85:e00991-16.
4. Coers J, Vance RE, Fontana MF, Dietrich WF. 2007. Restriction of *Legionella pneumophila* growth in macrophages requires the concerted action of cytokine and Naip5/Ipaf signalling pathways. Cell Microbiol 9:2344-57.
